# SEL1L plays a major role in human malignant gliomas

**DOI:** 10.1002/cjp2.134

**Published:** 2019-09-30

**Authors:** Marta Mellai, Laura Annovazzi, Renzo Boldorini, Luca Bertero, Paola Cassoni, Pasquale De Blasio, Ida Biunno, Davide Schiffer

**Affiliations:** ^1^ Dipartimento di Scienze della Salute, Scuola di Medicina Università del Piemonte Orientale “A. Avogadro” Novara Italy; ^2^ Fondazione Edo ed Elvo Tempia Valenta – ONLUS Biella Italy; ^3^ Ex Centro Ricerche/Fondazione Policlinico di Monza Vercelli Italy; ^4^ Dipartimento di Scienze Mediche Università degli Studi di Torino/Città della Salute e della Scienza Torino Italy; ^5^ ISENET Biobanking Milano Italy; ^6^ Istituto di Ricerca Genetica e Biomedica, Consiglio Nazionale delle Ricerche Milano Italy

**Keywords:** brain tumours, glioblastoma, SEL1L, microglia/macrophages, prognosis

## Abstract

*Suppressor of Lin‐12‐like (C. elegans)* (SEL1L) participates in the endoplasmic reticulum‐associated protein degradation pathway, malignant transformation and stem cell biology. We explored the role of SEL1L in 110 adult gliomas, of different molecular subtype and grade, in relation to cell proliferation, stemness, glioma‐associated microglia/macrophages (GAMs), prognostic markers and clinical outcome. SEL1L protein expression was assessed by immunohistochemistry and Western blotting. Genetic and epigenetic alterations were detected by molecular genetics techniques. SEL1L was overexpressed in anaplastic gliomas (World Health Organization [WHO] grade III) and in glioblastoma (GB, WHO grade IV) with the highest labelling index (LI) in the latter. Immunoreactivity was significantly associated with histological grade (*p* = 0.002) and cell proliferation index Ki‐67/MIB‐1 (*p* = 0.0001). In GB, SEL1L co‐localised with stemness markers Nestin and Sox2. Endothelial cells and vascular pericytes of proliferative tumour blood vessels expressed SEL1L suggesting a role in tumour neo‐vasculature. GAMs consistently expressed SEL1L. SEL1L overexpression was significantly associated with *TERT* promoter mutations (*p* = 0.0001), *EGFR* gene amplification (*p* = 0.0013), LOH on 10q (*p* = 0.0012) but was mutually exclusive with *IDH1/2* mutations (*p* = 0.0001). SEL1L immunoreactivity correlated with tumour progression and cell proliferation, conditioning poor patient survival and response to therapy. This study emphasises *SEL1L* as a potential biomarker for the most common subgroup of *TERT* mutant/*EGFR* amplified/*IDH‐*WT GBs.

## Introduction

Gliomas are the most common primary tumours of the central nervous system (CNS). According to the revised 2016 World Health Organization (WHO) classification of CNS tumours, they are classified into genetically and histologically identified entities and variants 1. Glioblastoma (GB) is the most aggressive tumour in adults, characterised by genotypic and phenotypic heterogeneity and by resistance to therapy. Despite major advances in treatment modalities, the prognosis of malignant gliomas remains dismal. GB has a median overall survival (OS) of 6 months after surgery, 12.1 months after radiotherapy (RT) and 14.6 months after RT plus concomitant and adjuvant chemotherapy (CHT) with temozolomide (TMZ) 2. The identification of new biomarkers involved in malignant transformation would be useful to improve diagnosis, prognosis and response to treatments.

*Suppressor of Lin‐12‐like protein (C. elegans)* (SEL1L) is a component of the unfolded protein response/endoplasmic reticulum‐associated degradation (UPR/ERAD) pathway 3, 4. UPR/ERAD pathway is the conserved protein quality‐control machinery of the ER that eliminates misfolded or unassembled proteins via the cytosolic ubiquitin proteasome system (UPS). SEL1L is an ER‐resident transmembrane adaptor protein for the E3 ubiquitin ligase hydroxymethylglutaryl reductase degradation protein 1 (HRD1). Within the ER, the SEL1L‐HRD1 complex is involved in the recruitment, retrotranslocation and ubiquitination of ERAD substrates 5, 6, 7.

Besides being an adaptive response, the UPR/ERAD pathway is triggered by cellular stress, hypoxia, reactive oxygen species and nutrient deprivation, commonly present in the tumour microenvironment (TME). Its constitutive activation has been described in several human malignancies 8, but its precise role is not fully clarified 9. However, SEL1L is implicated in tumour pathogenesis 10. *SEL1L* functions depend on tumour context being either down‐modulated in breast and pancreatic cancer 11, 12, or up‐modulated in prostate, lung and cervical cancers 13, 14, 15, including metastasis 16. In colorectal cancer, *SEL1L* is up‐regulated in the initial phases of neoplastic transformation, suggesting a potential function in tumour initiation and progression 17. In gliomas, SEL1L protein expression significantly increases with malignancy 18. Increased UPR activation, which correlates with tumour aggressiveness, has recently been observed in GB patients 19 where a single nucleotide polymorphism (rs12435998 within intron 3) conditions survival and response to TMZ‐based RT‐CHT 20.

Besides tumours, SEL1L participates in normal neurogenesis and in neurodegenerative diseases 10. It is involved in cell‐matrix interactions during neurogenesis, promotes cell cycle accumulation in G1 phase 21 and maintenance of a neural progenitor status and lineage determination 22. SEL1L also contributes to CNS differentiation and vasculogenesis by modulating Notch signalling 23, 24. UPR/ERAD and UPS pathways are significantly involved in regulating vascular smooth muscle (SM) cell plasticity, survival and phenotype 25. Murine SEL1L (mSEL‐1L) was shown to be expressed during CNS development 26 and its deficiency led to severe multi‐organ defects including brain 27. mSEL‐1L was also shown to have an essential function in guaranteeing the balance between self‐renewal and differentiation in neural progenitors 22, possibly by controlling self‐renewal and fate determinant regulators 18, 22.

This study aimed to investigate SEL1L expression in 110 adult human gliomas of different molecular subtype and grade using healthy nervous tissue as control. Here, we analysed the relationship of SEL1L with (1) tumour progression and cell proliferation, in association with TME; (2) stemness potential; (3) prognostic genetic and epigenetic markers (*IDH1/2*, *TERT* promoter and *TP53* mutations, *EGFR* gene amplification, *MGMT* promoter hypermethylation, 1p/19q co‐deletion, *ATRX* status, LOH on 9p, 10q and 17p); and (4) patient survival and response to therapy.

## Materials and methods

### Brain tumour specimens

A total of 110 adult glioma specimens were retrospectively collected from the archives of the Research Center/Policlinico di Monza Foundation (Vercelli, Italy) (see supplementary material, Table S1). Patients underwent partial or total resection at different Italian Neurosurgery Units. Surgical tumour samples were formalin fixed, paraffin embedded (FFPE) and cut in 5 μm‐thick sections. The histological diagnosis was in agreement with the current WHO guidelines 1. *IDH‐*mutant tumours with retained nuclear ATRX expression and classic oligodendroglial histology but with absence of total 1p/19q co‐deletion were classified as not otherwise specified (NOS) oligodendrogliomas 1.

Healthy nervous tissue was obtained from brain autopsy of patients who died for vascular encephalopathies.

Human cell lines (fetal cortex CB660 and GB‐derived GliNS2 and G144) were provided by ISENET Biobanking (Milan, Italy, http://www.isenet.it).

### Ethics statement

Human brain specimens were used in compliance with the ethical human subject principles of the World Medical Association Declaration of Helsinki Research. Written informed consent was obtained from patients after Institutional Ethics Committee approval.

### Patient stratification

Survival data were available for a subgroup of 58 patients and clinical follow‐up (FU) for 44 of them (18 low‐grade gliomas [LGGs] and 26 GBs). Twenty‐two *IDH*‐WT GBs received post‐operative fractionated RT (60 Gy total dose; 2 Gy × 5 days/week for 6 weeks) with concomitant CHT with TMZ (75 mg/m^2^/daily × 7 days/week for 6 weeks) followed by adjuvant TMZ (200 mg/m^2^ × 5 days/week every 4 weeks for 6–12 cycles) according to the EORT‐NCIC protocol regimen published by Stupp *et al* 2. Two GBs received only RT, whereas 2 cases only TMZ. Among diffuse gliomas, 1 *IDH*‐WT and 3 *IDH*‐mutant astrocytomas, 1 *IDH*‐mutant/1p19q‐codel and 4 NOS oligodendrogliomas were treated according to Stupp's protocol. The same regimen was applied to anaplastic oligodendrogliomas (2 *IDH*‐mutant/1p19q‐codel and 1 NOS). TMZ alone was administered to 4 diffuse *IDH*‐mutant astrocytomas and 2 NOS oligodendrogliomas. Patients with a Karnofsky performance score < 60 received best supportive care.

### *SEL1L* silencing by short interference

For silencing experiments, the human fetal CB660 cell line (1 × 10^6^ cells) was transiently nucleofected with 100 pmol small interference RNA (siRNA) against the 5′ end of the *SEL1L* coding sequence and non‐targeting siRNA (NT siRNA) (Ambion, Life Technologies, Monza, Italy) using the Nucleofector® and Amaxa nucleofector kit V (Lonza, Basel, Switzerland). Scrambled siRNAs were used in order to guarantee minimal or no off‐target activity and the reliability of the silenced phenotype.

### Immunohistochemistry

Immunohistochemistry (IHC) was performed using a Ventana Full BenchMark® XT automated immunostainer (Ventana Medical Systems Inc., Tucson, AZ, USA) and the ultraView™ Universal DAB Detection Kit (Ventana Medical Systems Inc.) as detection system. Heat‐induced epitope retrieval was obtained with Tris–EDTA, pH 8. Negative controls were obtained by omission of the primary antibody. Primary antibodies are listed in supplementary material, Table S2.

Double immunostaining for SEL1L/Ki‐67/MIB‐1, SEL1L/GFAP, SEL1L/IDH1^R132H^, SEL1L/Iba‐1, SEL1L/CD163, ATRX/Iba‐1 and CD34/SEL1L was performed using ultraView™ Universal Alkaline Phosphatase Red Detection Kit (Ventana Medical Systems Inc.).

Two overlapping antibodies against the N‐terminal region of SEL1L were used: a mouse monoclonal (MSel1) antibody (amino acids 1–285, exons 1–8) 28 and a rabbit polyclonal antibody (amino acids 159–186, exons 4–5) (#PA5‐24179, Thermo Fisher Scientific Inc., Walthman, MA, USA). Immunoreactivity was evaluated using a semi‐quantitative system for the percentage of positive cells and the staining intensity in 5 randomly selected microscopic high power fields (HPF) at ×400 magnification *per* tumour section. The staining extent was scored as 0 (<1%), 1 (1–25%), 2 (26–49%), 3 (50–74%) and 4 (≥75%) according to the percentage of positive cells. The staining intensity was classified into four grades: 0, absence of immunoreaction; 1, weak staining; 2, moderate positivity; 3, strong reactivity. In particular, we considered the nuclear (N), cytoplasmic (C) and total staining (N + C) of tumour cells. All cases were evaluated independently by 2 experienced pathologists (DS and RB). SEL1L+ glioma‐associated microglia/macrophages (GAMs) and myeloid cells were excluded from cell counts.

IHC for ATRX was used as surrogate marker for the mutation status of the *ATRX* gene.

### Protein extraction and Western blotting

Whole protein extracts from cell lines were isolated as reported 18. They were resolved by 10% SDS‐PAGE, transferred on PVDF membranes and probed with MSel1 anti‐SEL1L 28 and anti‐Vinculin specific antibodies (Sigma Aldrich Co., St. Louis, MO, USA) using X‐BlotP100 as hybridisation chamber (http://www.isenet.it). Proteins were detected by ECL assay (Genespin, Milan, Italy) and quantified using Scion Image program (http://www.scioncorp.com). Data were expressed as averages of three independent experiments.

Subcellular protein fractions of cell lines were obtained using the Subcellular Protein Fractionation Kit (Pierce Biotechnology, Rockford, IL, USA). Anti‐α‐tubulin and anti‐Sox2 antibodies (Millipore, Burlington, MA, USA) were used as controls for the cytosolic and nuclear fractions, respectively.

### Molecular genetics

Genomic DNA (gDNA) from FFPE tumour samples was isolated using the QIAamp DNA Mini Kit (Qiagen NV, Venlo, The Netherlands).

Search for mutations in *IDH1* (exon 4) (GenBank sequence NM_005896), *IDH2* (exon 4) (GenBank sequence NM_002168), *TERT* gene promoter region (GenBank accession no. NM_198253) and *TP53* (exons 4–8) genes (GenBank sequence NM_000546) was performed by Sanger direct sequencing on an ABI® 3130 Genetic Analyzer (Thermo Fisher Scientific Inc.) 29. Sequence variant nomenclature was in agreement with the current Human Genome Variation Society guidelines (http://varnomen.hgvs.org/).

The 1p/19q chromosomal status was assessed by multiplex ligation‐dependent probe amplification (MLPA) using the SALSA‐MLPA Kit P088‐C2 (MRC‐Holland, The Netherlands) 30.

Allelic imbalances on 9p, 10q and 17p chromosomes were detected by loss of heterozygosity (LOH) analysis and fragment analysis 31. The *EGFR* gene amplification status (GenBank accession no. NM_005228) was assessed as described 31.

Quantitative methylation specific‐PCR (qMS‐PCR) followed by capillary electrophoresis was used to determine the *MGMT* promoter hypermethylation status (GenBank accession no. NM_002412) 32.

### Statistical methods

Associations between categorical variables were evaluated using 2 × 2 contingency tables by the two‐tailed Fisher's exact test. The Pearson's correlation coefficient was used to analyse the relationship between SEL1L immunoreactivity and Ki‐67/MIB‐1 LI.

OS was defined as the time between histological diagnosis and patient death or last FU. Patient alive at last FU were considered as censored events. Survival curves were estimated using the Kaplan–Meier method and differences among groups were compared by the log‐rank test (Mantel–Cox). For survival analysis, cases were dichotomised into the following categories: low‐SEL1L expression cases (≤25% positive tumour cells, grades 0 and 1) and high‐SEL1L expression cases (>25% positive tumour cells, grades 2–4).

Analysis was carried out by SPSS v24.0 software (SPSS Inc., Chicago, IL, USA). *P* values <0.05 were considered as statistically significant.

## Results

### *SEL1L* silencing by siRNA

*SEL1L* silencing experiments on the human fetal CB660 cell line confirmed that *SEL1L* codes for at least three variants (AceView, http://www.ncbi.nlm.nih.gov/IEB/Research/Acembly/) (see supplementary material, Figure S1A–C). siRNA on *SEL1L* exon 12 and scrambled control (C) (panel A) showed the down‐modulation of the different SEL1L variants: 100 kDa isoform, corresponding to the ER‐resident variant SEL1LA (SEL1LA‐p100), 70/78 kDa isoform (SEL1L‐p70/80) with a predominant nuclear location, and 38 kDa secreted variant (SEL1L‐p38), generally expressed in tumour cells. Quantitative protein levels (panel B) from WB analysis indicated the decrease of the three isoforms compared to untreated (WT) and scrambled (C) samples. The nuclear localisation of the SEL1L‐p70/80 variant was revealed by the #PA5‐24179 antibody (panel C) upon protein subcellular fractionation.

### SEL1L expression in healthy nervous tissue

#### Cerebral cortex and white matter

The MSel1 antibody revealed a granular reaction in the cytoplasm of neurons and weakly positive cytoplasmic and perinuclear staining of glial cells, including perineuronal and perivascular oligodendroglial satellites (see supplementary material, Figure S2A). Intense nuclear immunostaining in glial cells and weak positivity in neurons were detected using the polyclonal #PA5‐24179 antibody (see supplementary material, Figure S2B).

In the white matter, most perifascicular and pericapillary oligodendrocytes showed nuclear and membranous immunopositivity with the MSel1 antibody (see supplementary material, Figure S2C). Ischaemic neurons showed enhanced immunoreactivity in their cytoplasm 18. The immunostaining intensity was weaker with the polyclonal #PA5‐24179 antibody (see supplementary material, Figure S2D). The few scattered ATRX+ nuclei corresponded to Iba1+ GAMs (see supplementary material, Figure S2E), whereas ATRX‐ nuclei to normal oligodendrocytes (see supplementary material, Figure S2F).

Endothelial cells (ECs) of normal blood vessels and capillaries did not reveal immunoreactivity with any of the antibodies.

#### Cerebellum

The MSel1 antibody revealed a granular reaction in the cytoplasm and dendrites of Purkinje cells (see supplementary material, Figure S2G). Most granule cells showed immunoreactivity in the nucleus, whereas glial cells of the molecular layer and white matter were weakly positive in the cytoplasm. The polyclonal #PA5‐24179 antibody showed weak cytoplasmic immunostaining of Purkinje cells (see supplementary material, Figure S2H). Some granule cells were variably and weakly positive in the nucleus. Glial cells of the molecular layer and white matter were weakly positive in the nucleus.

#### Brain stem

SEL1L expression was confined to the ependymal layer (in the nucleus and cytoplasm) with both antibodies.

### SEL1L expression in human gliomas

A total of 110 grade I–IV gliomas were studied using both mono‐ and polyclonal anti‐N‐SEL1L antibodies. SEL1L was expressed in the cytoplasm and/or in the nucleus of tumour cells, according to the antibody used, in GAMs and myeloid cells.

**Figure 1:**
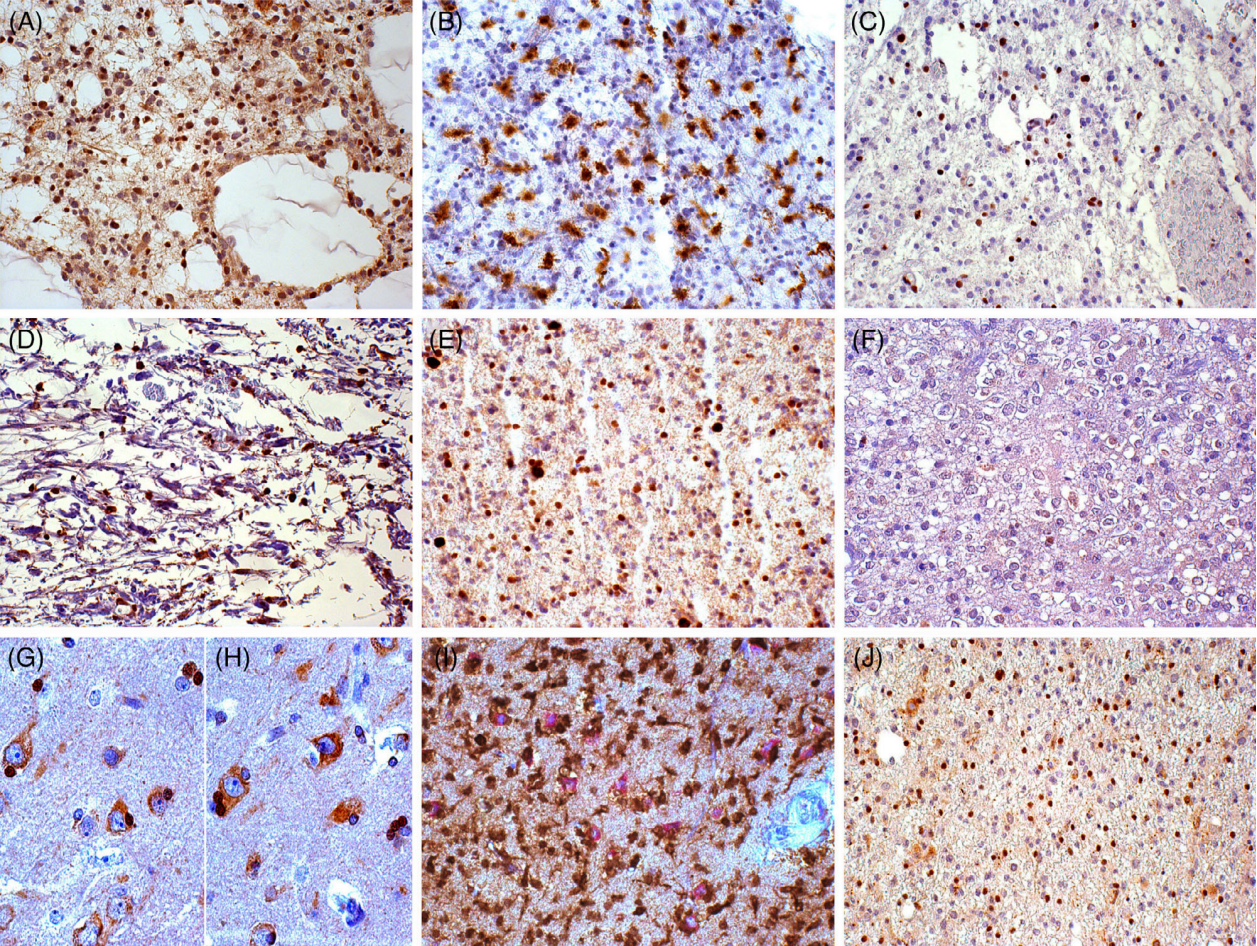
Immunohistochemistry in low‐grade gliomas using the monoclonal anti‐SEL1L antibody. (A) Diffuse astrocytoma, SEL1LA+ microglia/macrophages; DAB, original magnification (OM) × 200. (B) Diffuse astrocytoma, Iba‐1+ microglia/macrophages; DAB, OM × 200. (C) Diffuse astrocytoma, ATRX‐ tumour cells and ATRX+ microglia/macrophages; DAB, OM × 200. (D) Pilocytic astrocytoma, SEL1LA+ microglia/macrophages; DAB, OM × 200. (E) Diffuse oligodendroglioma, ATRX+ tumour cells and ATRX+ microglia/macrophages; DAB, OM × 200. (F) Diffuse oligodendroglioma with honeycomb appearance, SEL1LA+ and SEL1LA‐ tumour cells; DAB, OM × 200. (G) and (H) Diffuse oligodendroglioma with honeycomb appearance, SEL1LA+ (G) and SEL1LA− (H) perineuronal satellites; DAB, OM × 400. (I) Perineuronal satellites, IDH1^R132H^+ tumour cells and SEL1LA+ neurons; double immunohistochemistry with DAB and Alkaline Phosphatase Red, respectively, OM × 200. (J) Perineuronal satellites, oligodendroglial infiltration, SEL1LA+ neurons and microglia/macrophages; DAB, OM × 200. DAB, 3,3′‐diaminobenzidine.

In diffuse astrocytomas, MSel1 antibody was weakly and variably expressed in the cytoplasm and in the perinuclear membrane of tumour cells (up to 15–20%) (Figure 1A). The few scattered SEL1L+ cells were identified as Iba‐1+ GAMs (Figure 1B,C). In pilocytic astrocytomas, SEL1L expression was never detected with both antibodies (Figure 1D). In diffuse ATRX+ oligodendrogliomas, no more than 5–10% of tumour cells showed variably positive nuclear, perinuclear and cytoplasmic staining (Figure 1E,F). Typical honeycomb appearance and perineuronal satellites variably showed either SEL1L+ or SEL1L− stained nuclei (Figure 1G,H). Neurons, intermingled with tumour parenchyma or in infiltration areas, and GAMs expressed SEL1L (Figure 1I,J).

**Figure 2:**
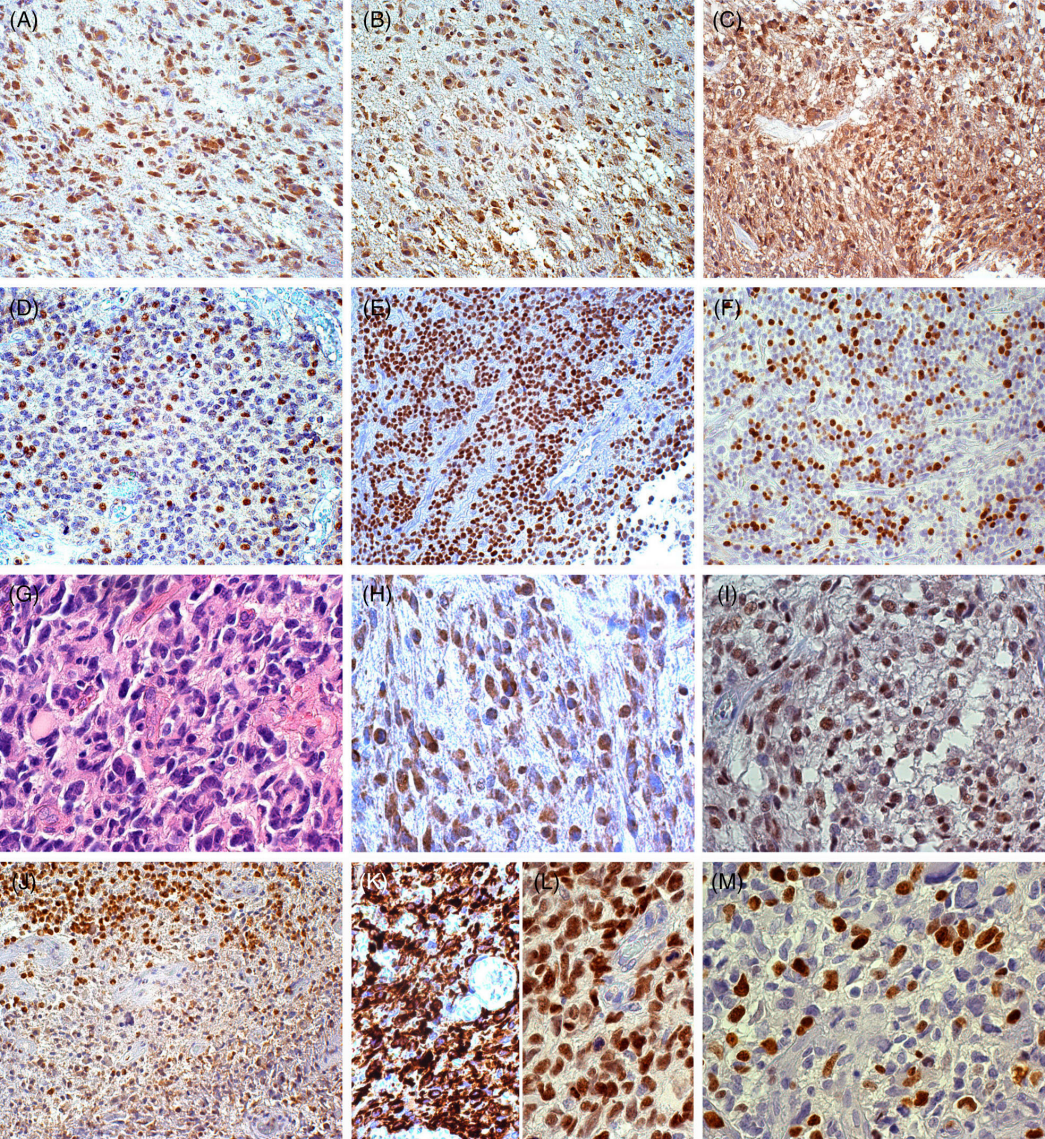
Immunohistochemistry in malignant gliomas with monoclonal and polyclonal anti‐SEL1L antibodies. (A) Anaplastic astrocytoma, SEL1LA+ neurons, microglia/macrophages and mostly tumour cells; DAB, original magnification (OM) × 200. (B) Anaplastic astrocytoma, SEL1L‐p70/80+ neurons, microglia/macrophages and mostly tumour cells; DAB, OM × 200. (C) Anaplastic oligodendroglioma, SEL1LA+ tumour cells in the cytoplasm and nucleus; DAB, OM × 200. (D) Anaplastic oligodendroglioma, SEL1L‐p70/80+ tumour cells in the nucleus; DAB, OM × 200. (E) Anaplastic oligodendroglioma, SEL1LA+ tumour cells; DAB, OM × 200. (F) Anaplastic oligodendroglioma, Ki‐67/MIB‐1+ cells; DAB, OM × 200. (G) GB, H&E; DAB, OM × 400. (H) GB, SEL1LA+ tumour cells in the cytoplasm and nuclei; DAB, OM × 400; (I) GB, SEL1L‐p70/80+ tumour cells in the nucleus; DAB, OM × 400. (J) GB, SEL1L‐p70/80+ tumour and myeloid cells around a perinecrotic pseudopalisade; DAB, OM × 200. (K) GB, Nestin+ tumour cells; DAB, OM × 400. (L) GB, Sox2+ tumour cells; DAB, OM × 400. (M) GB, Ki‐67/MIB‐1+ cells; DAB, OM × 400. DAB, 3,3′‐diaminobenzidine.

In anaplastic gliomas, the frequency of SEL1L+ tumour cells ranged from >25 to 70–80% as did the immunostaining intensity, with a predominant nuclear staining (Figure 2A–F). In GBs, diffuse and variable SEL1L immunoreactivity was found roughly in 100% tumour cells (Figure 2G–I) in the cytoplasm and/or in the nucleus, in relation to the antibody used. Hyperproliferative and hyperangiogenic areas, perinecrotic pseudopalisades (Figure 2J) and the inner layer of perivascular cuffings of co‐option areas displayed the highest SEL1L LI. In these areas, SEL1L+ tumour cells corresponded to Nestin+ and Sox2+ cells (Figure 2K,L). The more intense nuclear immunostaining, revealed by the polyclonal #PA5‐24179 antibody, corresponded to Ki‐67/MIB‐1+ nuclei and to mitoses (Figure 2M). The diffuse background staining in GBs could be related to the secreted variant SEL1L‐p38.

The immunostaining pattern detected by the polyclonal #PA5‐24179 antibody was mainly nuclear in all molecular subtypes.

Reactive astrocytes displayed weak cytoplasmic SEL1L expression.

### Relationship of SEL1L expression with tumour neo‐vasculature

**Figure 3:**
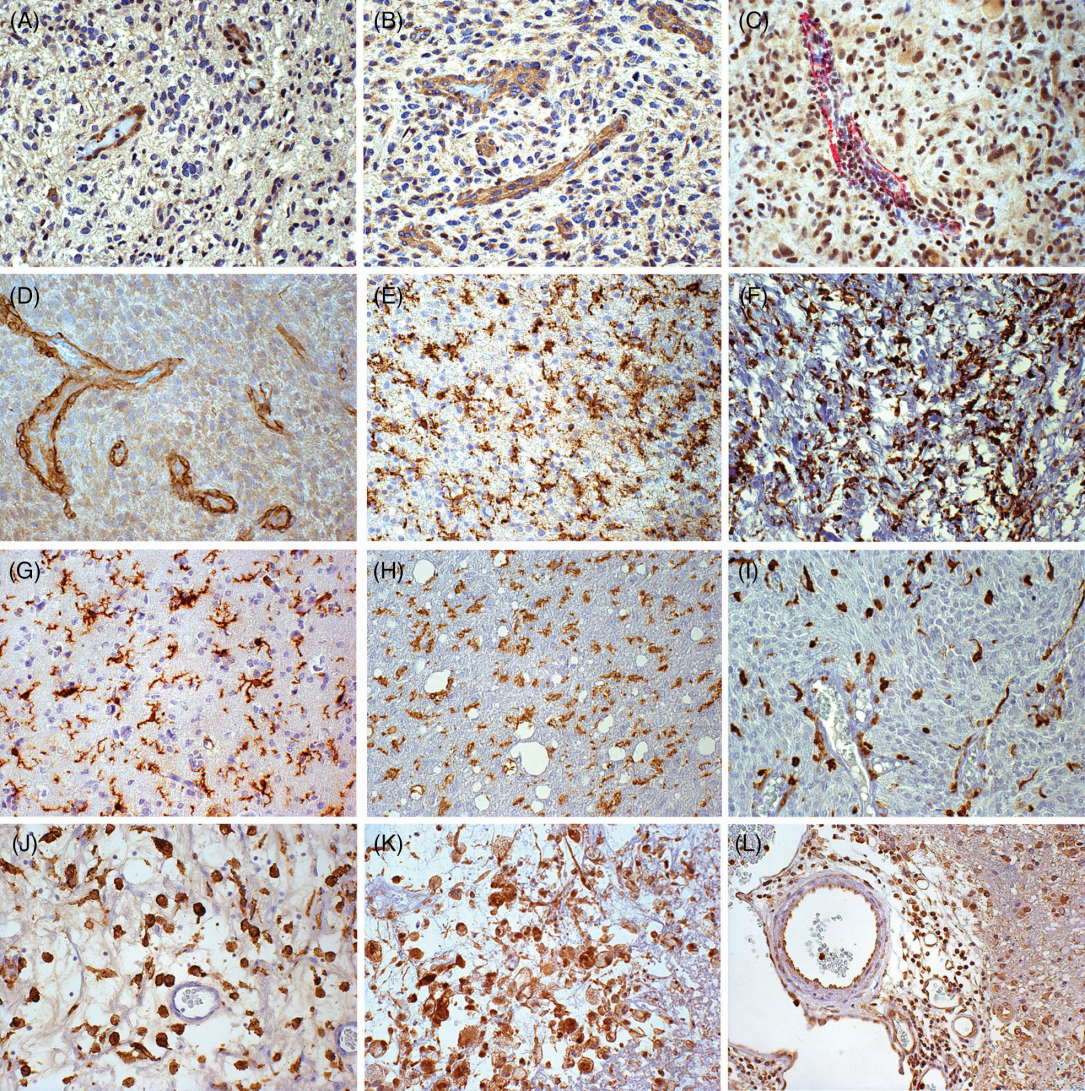
Immunohistochemistry using monoclonal and polyclonal anti‐SEL1L antibodies with regard to tumour neo‐vasculature and GAMs. (A) GB with proliferated tumour blood vessels, SEL1LA+ ECs and vascular pericytes; DAB, original magnification (OM) × 200. (B) GB with proliferated tumour blood vessels, SEL1L‐p70/80+ ECs and pericytes; DAB, OM × 200. (C) GB with proliferated tumour blood vessels, SEL1LA+ and α‐SM‐Actin+ vascular pericytes; double immunohistochemistry with DAB and Alkaline Phosphatase Red, respectively, OM 200×. (D) GB with proliferated tumour blood vessels, NG2/CSPG4+ ECs and pericytes; DAB, OM × 200. (E) Diffuse astrocytoma, Iba‐1+ GAMs; DAB, OM × 200. (F) Pilocytic astrocytoma, Iba‐1+ GAMs; DAB, OM × 200. (G) Diffuse oliodendroglioma, Iba‐1+ GAMs; DAB, OM × 200. (H) Anaplastic astrocytoma, Iba‐1+ GAMs; DAB, OM × 200. (I) Anaplastic oligodendroglioma, Iba‐1+ GAMs; DAB, OM × 200. (J) GB, Iba‐1+ scattered macrophages in tumour parenchyma; DAB, OM × 200. (K) GB, SEL1LA+ scattered macrophages in tumour parenchyma; DAB, OM × 200. (L) GB, SEL1L‐p70/80+ peri‐ and intravascular macrophages; DAB, OM × 200. DAB, 3,3′‐diaminobenzidine.

SEL1L was not expressed in ECs and vascular pericytes of normal blood vessels or quiescent tumour blood vessels of both LGGs and high‐grade gliomas (HGGs). Conversely, ECs and vascular pericytes of neo‐formed proliferated tumour vessels (MVPs and glomeruli) intensely expressed SEL1L in the nucleus and cytoplasm (Figure 3A,B). Vascular pericytes were revealed by α‐smooth muscle actin (α‐SMA) and neuron glial antigen 2/chondroitin sulphate proteoglycan 4 (NG2/CSPG4) immunostaining (Figure 3C,D).

### Relationship of SEL1L expression with GAMs

GAMs, as revealed by Iba‐1, CD68 and CD163 immunostaining, showed a similar distribution and frequency in LGGs and HGGs. Whereas Iba‐1+/CD163‐ cells prevailed in the former, CD163^+^ cells were prevalent in the latter.

GAMs showed positive cytoplasmic and nuclear immunostaining using the MSel1 antibody, as detected by double immunostaining for SEL1L/Iba‐1/CD68/CD163. In LGGs, microglia cells corresponded to scattered SEL1L+ cells (Figure 3E–I). In HGGs, the diffuse SEL1L+ staining in hyperproliferative areas overlapped the expression of GAMs and Ki‐67/MIB‐1+ nuclei. In GBs, parenchymal tumour areas enriched in Iba‐1+/CD163+ cells were also enriched in SEL1L+ cells (Figure 3J,K). Peri‐ and intravascular macrophages were intensely SEL1L+ in the nucleus (Figure 3L).

### Molecular genetics

*TERT* promoter mutation was detected in 34 of 56 (60.7%) gliomas. The mutation rate was 26 of 31 (83.9%) in *IDH*‐WT GBs and 8 of 25 (32%) in LGGs. The point mutation c.−124C>T (C228T) accounted for 24 of 34 (70.6%) and the c.−146C>T (C250T) mutation for 10 of 34 (29.4%).

Point mutations at codon Arg132 of the *IDH1* gene were identified in 21 of 62 (33.9%) gliomas. The missense mutation c.395G>A (p.Arg132His) accounted for 19 of 21 (90.5%), c.394C>A (p.Arg132Ser) and c.394C>G (p.Arg132Gly) for 1 of 21 (4.8%) each. No mutation was detected in the *IDH2* gene at codon Arg172.

The mutation rate of the *TP53* gene in astrocytic gliomas was 9 of 43 (20.9%), 4 of them showing LOH on the chromosome arm 17p. All mutations were somatic changes.

*MGMT* promoter hypermethylation was detected by MS‐qPCR in 19 of 43 (44.2%) tumours. *EGFR* gene amplification occurred in 19 of 60 (31.7%) gliomas.

Malignant gliomas showed LOH on 10q in 16 of 18 (88.9%) cases and LOH on 9p in 8 of 18 (44.4%) cases. LOH on 17p was detected in 4 of 17 (23.5%) astrocytic gliomas.

Finally, *IDH*‐WT GBs, oligodendroglial tumours and pilocytic astrocytomas all retained ATRX protein expression in the nucleus. Conversely, it was lost in 10 of 12 (83.3%) astrocytic LGGs and in the *IDH*‐mutant GB.

### Associations with clinical and molecular features

**Figure 4:**
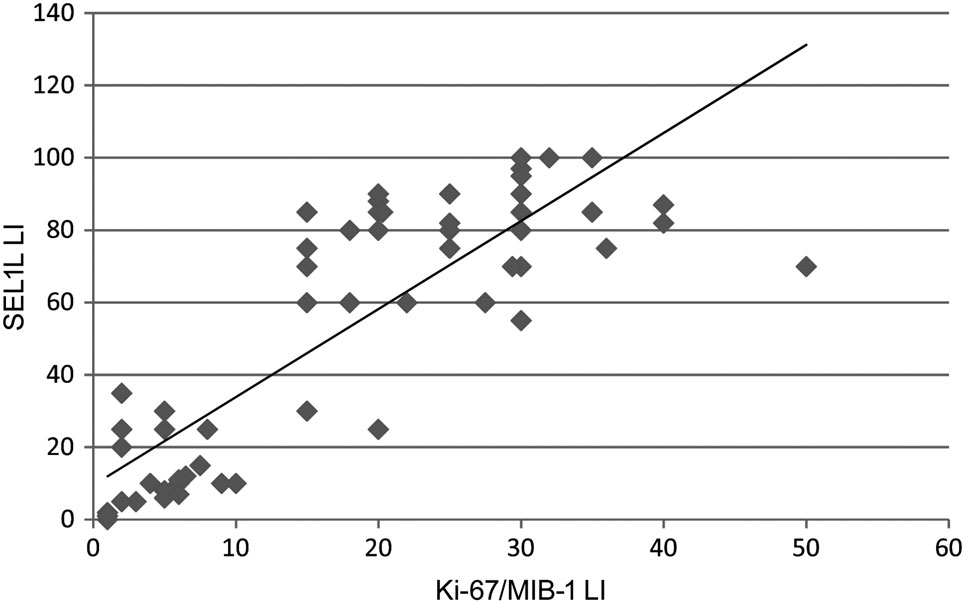
Correlation between SEL1L and Ki‐67/MIB‐1 LIs. Linear regression analysis between SEL1L immunoreactivity and Ki‐67/MIB‐1 LI (*ρ* = 0.797, *p* = 0.0001).

SEL1L immunoreactivity assessed by the MSel1 antibody was significantly associated with histological malignancy grade (*p* = 0.002). Nuclei with the highest SEL1L staining intensity statistically matched, in terms of distribution, Ki‐67/MIB‐1+ nuclei (Pearson correlation coefficient *ρ* = 0.797, *p* = 0.0001) (Figure 4).

No significant associations were found with patient age, gender or anatomical location.

SEL1L immunoreactivity was significantly associated with *TERT* promoter mutation (*p* = 0.0001), *EGFR* gene amplification (*p* = 0.0013), LOH on 10q (*p* = 0.0012) and (borderline) with LOH on 9p (*p* = 0.0546). Conversely, SEL1L overexpression was mutually exclusive with the presence of *IDH1/2* mutations (*p* = 0.0001) (see supplementary material, Table S3).

### Survival analysis

**Figure 5:**
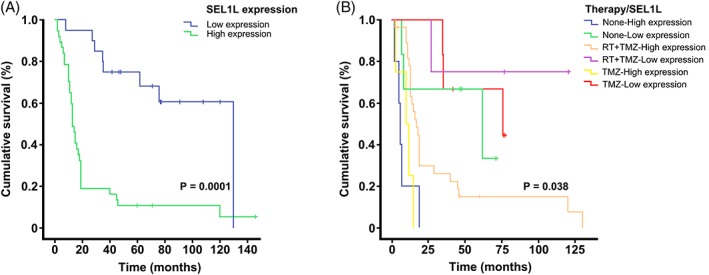
Relationship between SEL1L expression and survival in glioma patients. (A) Kaplan–Meier survival curves for OS in 58 glioma patients with respect to SEL1L immunoreactivity using the MSel1 antibody. (B) Kaplan–Meier survival curves for OS in 44 glioma patients after patient stratification for post‐surgical treatment.

The relationship between SEL1L protein expression and OS was analysed in a subgroup of 58 patients, including 2 pilocytic astrocytomas, 20 diffuse and 6 anaplastic gliomas, and 30 GBs. By Kaplan–Meier survival analysis, SEL1L immunoreactivity was significantly associated with a worse prognosis (*p* = 0.0001) (Figure 5A). The median survival time was 130 months for cases with low‐SEL1L expression and 13 months for cases with high‐SEL1L expression. Censored cases were 12 of 20 (60%) for the former and 4 of 38 (10.5%) for the latter. Moreover, in the subgroup of 44 patients with recorded FU, SEL1L overexpression conditioned a poor response to the post‐surgical therapy (*p* = 0.038) (Figure 5B).

## Discussion

In human malignancies, SEL1L plays either an oncogenic or a tumour suppressive role according to the cellular context or the isoform predominance. At the protein level, SEL1L may have a double cellular location (nucleus and cytoplasm) possibly related to its variable response to genotoxic insults 14, 17. In the present series of gliomas, SEL1L cellular location was assessed using two overlapping antibodies against the N‐terminal region of the protein that revealed different variants. The detection of the cytoplasmic variant prevailed over the nuclear variant using the MSel1 antibody, whereas the #PA5‐24179 antibody mainly revealed the latter. The subcellular location corresponds to three variants demonstrated by siRNA experiments on the human fetal CB660 cell line and on GB‐derived cell lines (as described in AceView) 33. The diffuse background reaction in GB specimens observed using both antibodies may be ascribed to the secreted variant SEL1L‐p38 33. The cytoplasmic and nuclear variants may exert the same biological function in different cellular compartments. The function of SEL1LA‐p100 could predominate in the cytoplasmic UPR/ERAD/UPS pathway in response to the exposure of tumour cells to intrinsic (genomic instability, oncogene expression and increased metabolic burden) and extrinsic (hypoxia, oxidative stress and nutrient deprivation) stresses within the TME. On the other hand, the nuclear SEL1L‐p70/80 variant might be involved in the maintenance of nuclear proteostasis in order to preserve the nuclear UPS from stress‐induced dysfunction 34. As a matter of fact, the *SEL1L* gene contains a nuclear localisation signal at the N‐terminal region of the protein 33. The presence of distinct proteostasis circuits, which cooperate in the quality control of proteins between nucleus and cytoplasm, has recently been reported 35, 36, 37, 38. A recent study also revealed a specific role for the ubiquitin‐like molecule NEDD8 in the defense mechanism against proteotoxic stess 39.

In healthy cerebral cortex and white matter, SEL1L is expressed in neurons and glial cells. In the cerebellum it is detected in cells of the molecular layer and in Purkinje cells. In brain stem, SEL1L is expressed in cells of the ependymal layer. The variable expression in normal oligodendrocytes of the white matter, perineuronal satellites and cerebellum granule cells may be related to their different functional status.

Our findings in gliomas are based on the immunohistochemical detection of SEL1L with both antibodies. The interpretation of the immunostaining should take into account the dual meaning of SEL1L, as a marker associated with either tumour progression or cell differentiation. Whereas SEL1L is not expressed or barely detectable in LGGs, HGGs exhibit significantly increased immunoreactivity, in terms of number of positive cells and reaction intensity. In GBs, SEL1L expression displays the highest LI in hyperproliferative areas, correlating with cell proliferation (Ki‐67/MIB‐1) and mitotic indices. In these areas, SEL1L immunostaining corresponds to the positivity of stemness markers, such as Nestin and Sox2. This finding is even more evident with the polyclonal antibody, which mainly reveals the nuclear immunoreactivity. In GBs, the cytoplasmic and nuclear staining varies consistently with the heterogeneous presence of undifferentiated and regressive areas. Hyperproliferative areas of GB are composed of GB stem cells (GSCs) or progenitors at variable differentiation stages 40, 41, 42, 43. In our opinion, GSCs and progenitors represent a functional status regulated by the TME 43, 44, 45, 46 rather than a cell type 43. This hypothesis is supported by the variable SEL1L expression observed in GB‐derived human cell lines (I. Biunno, unpublished data). On the other hand, SEL1L might contribute to tumour growth and resistance to therapy 47.

The weak immunoreactivity in the cytoplasm of reactive astrocytes revealed by the MSel1 antibody is in line with the differentiation potential of SEL1L and the poor positivity of astroglia in the healthy nervous tissue.

The constitutive SEL1L expression in GAMs hampers the evaluation of its expression from tumour cells. In HGGs, GAMs are mainly Iba‐1+/CD163+/CD45^high^, that is, corresponding to blood‐borne macrophages. In LGGs and in infiltration areas of HGGs, Iba‐1+/CD163‐/CD45^low^ GAMs prevail as reactive ramified microglia deriving from resident microglia 48, 49. As the frequency of GAMs is similar in LGGs and HGGs, the increased SEL1L expression in the latter can be attributed to the increased number of SEL1L+ tumour cells and to their staining intensity. The debated question on GAMs, that is, if they are ‘friends or foes’ in respect to the tumour, has been solved, considering GAMs as favouring tumour progression 48, 49, 50, 51. However, uncertainties still remain, mainly in relation to the timing of the blood–brain barrier disruption and the origin of GAMs 48, 49, 52. As a matter of fact, the scattered SEL1L+ cells in pilocytic astrocytoma correspond to Iba‐1+ GAMs.

SEL1L is not detected in ECs of normal or quiescent tumour blood vessels, but it is strongly expressed by ECs and vascular pericytes of the proliferative tumour neo‐vasculature in malignant gliomas (microvascular proliferations and glomeruli). This is of particular importance with reference to pericytes because SEL1L influences development of the vascular network during embryogenesis and pericyte plasticity 23, 24. SEL1L detection in pericytes may be attributed to its expression by GSCs within the perivascular niche 53, 54, 55, 56.

In the present study, SEL1L overexpression associates significantly with *TERT* promoter mutation, *EGFR* gene amplification, LOH on 10q and (borderline) 9p chromosomes, all well‐known negative prognostic markers for gliomas. By contrast, SEL1L correlates inversely with *IDH1/2* mutations that are the most relevant prognostic marker. As the above mentioned genetic profile is commonly detected in the molecular subgroup of IDH‐WT GBs, SEL1L may be suggested as a new potential biomarker for these tumours.

In agreement with the above findings, SEL1L overexpression conditions an unfavourable prognosis in glioma patients and a worse response to TMZ‐based RT‐CHT. As a matter of fact, current therapeutic treatments for GB patients induce ER proteostasis imbalance that might, ultimately, contribute to the selection of an adapted cell population that is resistant to the initial treatment 57. In line with our results, SEL1L is an important mediator for TMZ resistance and a poor prognostic factor for GB patients 58. Recently, using a CRISPR‐Cas9 approach in GB‐derived cell lines, actionable pathways including regulator of stemness and stress response (ufmylation and ERAD pathways) have been identified as responsible for tumour growth and TMZ sensitivity 59. In particular, SEL1L and HRD1 were among the highly ranked GB‐specific fitness genes in GSCs emphasising the relevance of proteostasis gene networks as a potential therapeutic strategy for GB.

All these findings support the involvement of SEL1L, as a member of the ER protein quality‐control machinery, in glioma progression, neo‐angiogenesis affecting TME (both immune cells and ECs), and in modulating efficacy of therapies.

## Author contributions statement

DS and IB planned the project and the study design. RB and PC contributed to tumour sample collection. MM performed literature research, data collection and interpretation, and drafted the paper. LA performed the experiments and contributed to text and figure design. LB assisted with the experiments and data analysis. DS, IB and MM edited the final manuscript. DS, IB and PD provided funding. All authors discussed and approved the submitted manuscript.

## Supporting information


**Figure S1.** SEL1L silencing by siRNAi and Western blotting analysis

**Figure S2.** Immunohistochemistry using monoclonal and polyclonal anti‐SEL1L antibodies in healthy nervous tissue

**Table S1.** Patient demographics
**Table S2.** List of primary antibodies used for immunohistochemistry
**Table S3.** Associations of SEL1L immunoreativity with common prognostic markers in gliomas
