# Supplementary figures and images for: SEL1L plays a major role in human malignant gliomas
Source: J Pathol Clin Res. 2019 Sep 30;6(1):17–29. doi: 10.1002/cjp2.134 (PMC6966709; doi:10.1002/cjp2.134)

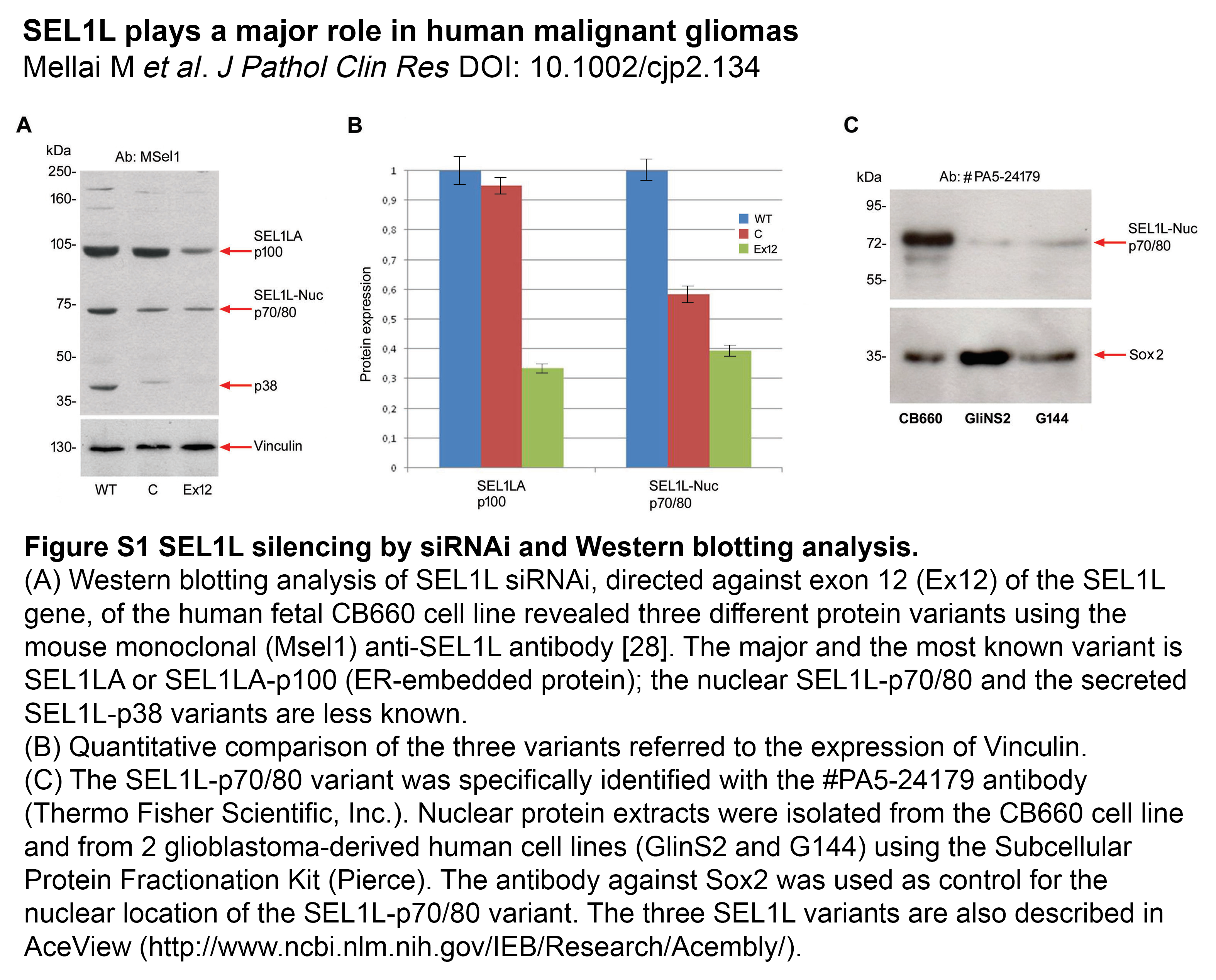

Supplement: Supplementary file 1 — Figure S1. SEL1L silencing by siRNAi and Western blotting analysis [file CJP2-6-17-s001.tif]

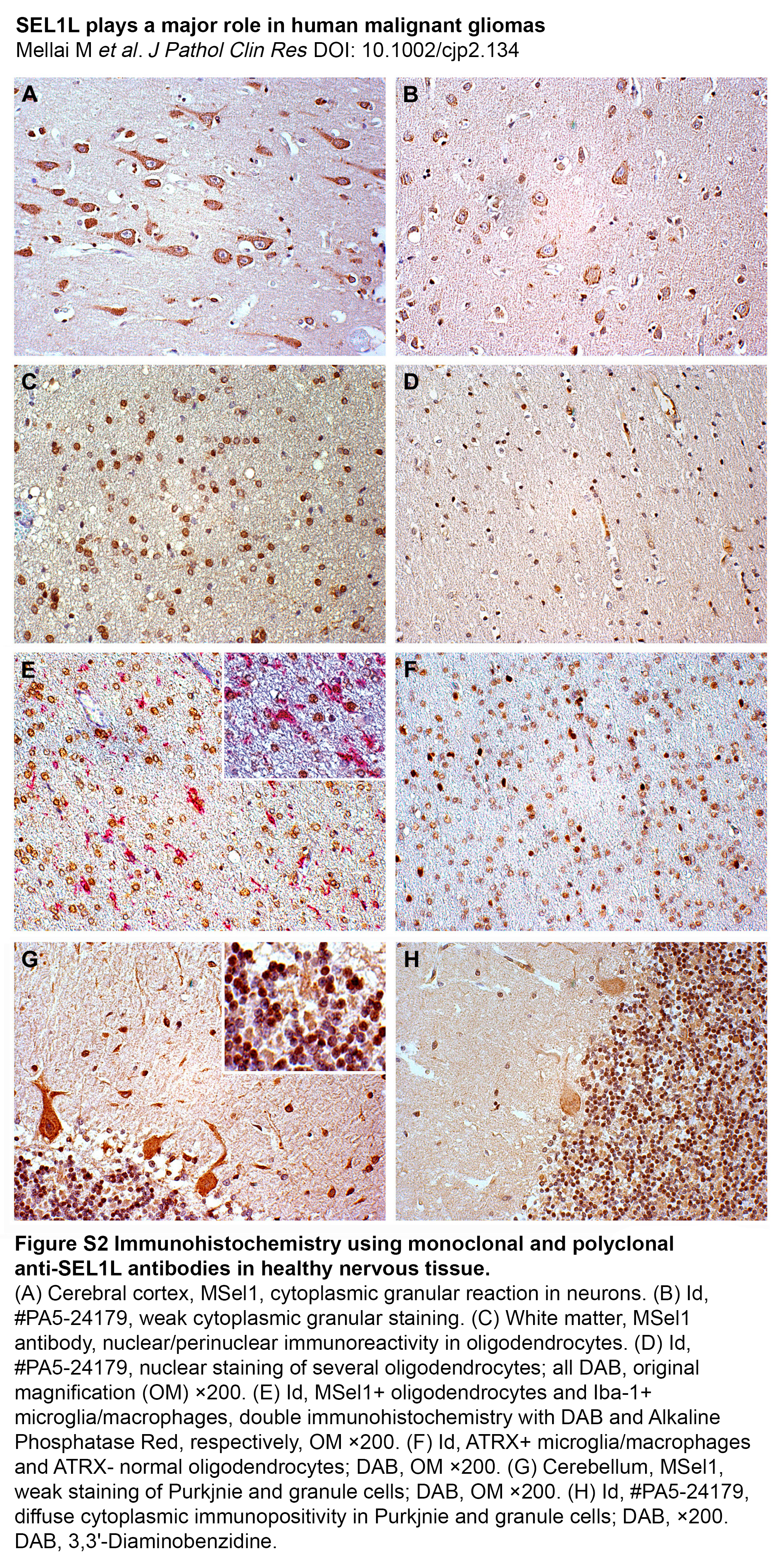

Supplement: Supplementary file 2 — Figure S2. Immunohistochemistry using monoclonal and polyclonal anti‐SEL1L antibodies in healthy nervous tissue [file CJP2-6-17-s002.tif]
