# Supplementary material for: SEL1L plays a major role in human malignant gliomas
Source: J Pathol Clin Res. 2019 Sep 30;6(1):17–29. doi: 10.1002/cjp2.134 (PMC6966709; doi:10.1002/cjp2.134)
Supplement: Supplementary file 3 — Table S1. Patient demographics Table S2. List of primary antibodies used for immunohistochemistry Table S3. Associations of SEL1L immunoreativity with common prognostic markers in gliomas [file CJP2-6-17-s003.docx]

**SEL1L plays a major role in human malignant gliomas**

Mellai M *et al. J Pathol Clin Res* DOI: 10.1002/cjp2.134

**Table S1** Patient demographics (*N* = 110)

| Tumor type | WHO  grading | Patients  (*n*) | Gender  (M/F) | Mean age (years) and range |
| --- | --- | --- | --- | --- |
| PA | I | 4 | 1/3 | 38 (19-51) |
| A, IDH-mutant | II | 12 | 5/7 | 42 (23-68) |
| A, IDH-wild type | II | 1 | 1/0 | 45 (25-52) |
| AA, IDH-mutant | III | 1 | 1/0 | 49 (24-75) |
| AA, IDH-wild type | III | 3 | 1/2 | 60 (23-83) |
| GB, IDH-wild type | IV | 57 | 25/32 | 60 (23-83) |
| GB, IDH-mutant | IV | 1 | 1/0 | 56 |
| O, IDH-mutant/1p19q co-deleted | II | 11 | 8/3 | 47 (26-79) |
| AO, IDH-mutant/1p19q co-deleted | III | 3 | 2/1 | 55 (31-80) |
| O, NOS | II | 6 | 3/3 | 55 (31-80) |
| AO, NOS | III | 8 | 6/2 | 26 (8-39) |

WHO, World Health Organization; PA, pilocytic astrocytoma; A, astrocytoma; AA, anaplastic astrocytoma; GB, glioblastoma; O, Oligodendroglioma; AO, anaplastic oligodendroglioma; IDH, isocitrate dehydrogenase; 1p19q co-deleted, 1p/19q co-deletion; NOS, not otherwise specified.

**SEL1L plays a major role in human malignant gliomas**

Mellai M *et al. J Pathol Clin Res* DOI: 10.1002/cjp2.134

**Table S2** List of primary antibodies used for immunohistochemistry

| Antibody (clone) | Source | Dilution | Code | Company |
| --- | --- | --- | --- | --- |
| N-SEL1L (MSel1)* | Mouse | 1: 350 | − | Kind gift |
| N-SEL1L (Polyclonal)* | Rabbit | 1: 50 | #PA5-24179 | Thermo Fisher Scientific, Inc. |
| Ki-67 (MIB-1)* | Mouse | 1 : 100 | M7240 | Dako |
| GFAP | Mouse | 1 : 200 | M0761 | Dako |
| IDH1RI32H (H09)* | Mouse | 1 : 20 | DIA H09 | Dianova |
| ATRX* | Rabbit | 1: 400 | HPA001906 | Sigma-Aldrich |
| CD34* | Mouse | Pre-diluted | 790-2927 | Ventana Medical Systems, Inc. |
| Iba-1* | Rabbit | 1 : 500 | #019-19741 | Wako Chemicals |
| CD68 (KP-1)* | Mouse | Pre-diluted | 790-2931 | Ventana Medical Systems, Inc. |
| CD163 (MRQ-26)* | Rabbit | Pre-diluted | 760-4863 | Ventana Medical Systems, Inc. |
| Nestin | Mouse | 1 : 200 | MAB5326 | Dako |
| Sox2* | Mouse | 1 : 100 | MAB2018 | R&D Systems |
| NG2/CSPG4 | Rabbit | 1: 50 | NBP1-89682 | Novus Biological |
| α-SMA (1A4)* | Mouse | Pre-diluted | 760-2833 | Ventana Medical Systems, Inc. |

GFAP, glial fibrillary acidic protein; IDH1, isocitrate dehydrogenase 1; ATRX, alpha-thalassemia/mental retardation syndrome X-linked; Iba-1, ionized calcium-binding adapter molecule 1; SOX2, sex determining region Y-box 2; NG2/CSPG4, neuron glial antigen 2/chondroitin sulphate proteoglycan 4; α-SMA, -smooth muscle actin.

* Heat-induced antigen retrieval.

**Table S3** Association of SEL1L immunoreactivity with common prognostic markers in gliomas

| Molecular marker | Overall mutation rate  (*n*, %) | Mutation rate in cases with SEL1L-low  expression  (*n*, %) | Mutation rate in cases with  SEL1L-high  expression  (*n*, %) | *P*-value |
| --- | --- | --- | --- | --- |
| TERT promoter mutations | 34/56 (60.7) | 3/16 (18.8) | 31/40 (77.6) | 0.0001 |
| EGFR amplification | 19/60 (31.7) | 1/20 (5) | 18/40 (45) | 0.0013 |
| LOH 9p | 15/43 (34.9) | 4/21 (19) | 11/22 (50) | 0.0546 |
| LOH 10q | 18/27 (66.7) | 2/9 (22.2) | 16/18 (88.9) | 0.0012 |
| IDH1/2 mutations | 21/62 (33.9) | 15/22 (68.2) | 6/40 (15) | 0.0001 |
| MGMT promoter methylation | 19/43 (44.2) | 2/6 (33.3) | 17/37 (45.9) | NS |
| 1p/19q co-deletion | 4/52 (7.7) | 1/14 (7.1) | 3/38 (7.9) | NS |
| LOH 17p | 4/17 (23.5) | 0/1 (0) | 4/16 (25) | NS |
| TP53 mutations | 9/43 (20.9) | 2/18 (11.1) | 7/25 (28) | NS |

SEL1L, suppressor of Lin-12-like (*C. elegans*);TERT, telomerase reverse transcriptase; EGFR, epidermal growth factor receptor; LOH, loss of heterozygosity; IDH, isocitrate dehydrogenase; MGMT, O6-Methylguanine-DNA methyltransferase;TP53, tumours protein p53; NS, not significant.
